# Supplementary material for: AI-based analysis of oral lesions using novel deep convolutional neural networks for early detection of oral cancer
Source: PLoS One. 2022 Aug 24;17(8):e0273508. doi: 10.1371/journal.pone.0273508 (PMC9401150; doi:10.1371/journal.pone.0273508)
Supplement: S1 Fig — (PDF) [file pone.0273508.s001.pdf]

S1 Fig. The receiver operating characteristic (ROC) curve of high performance CNN-based multiclass classification models: (a) DenseNet-169, (b) ResNet-101.

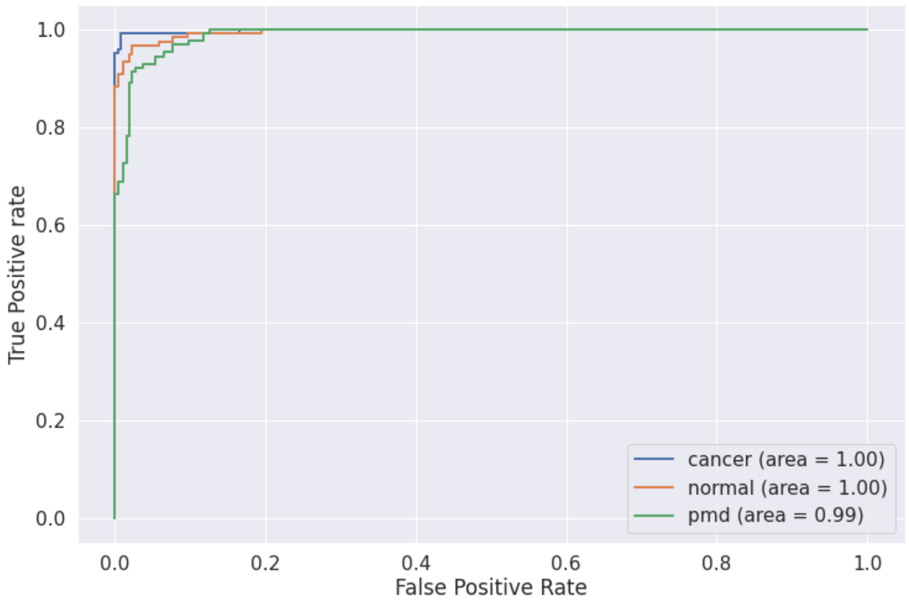

(a) ROC curve of DenseNet-169

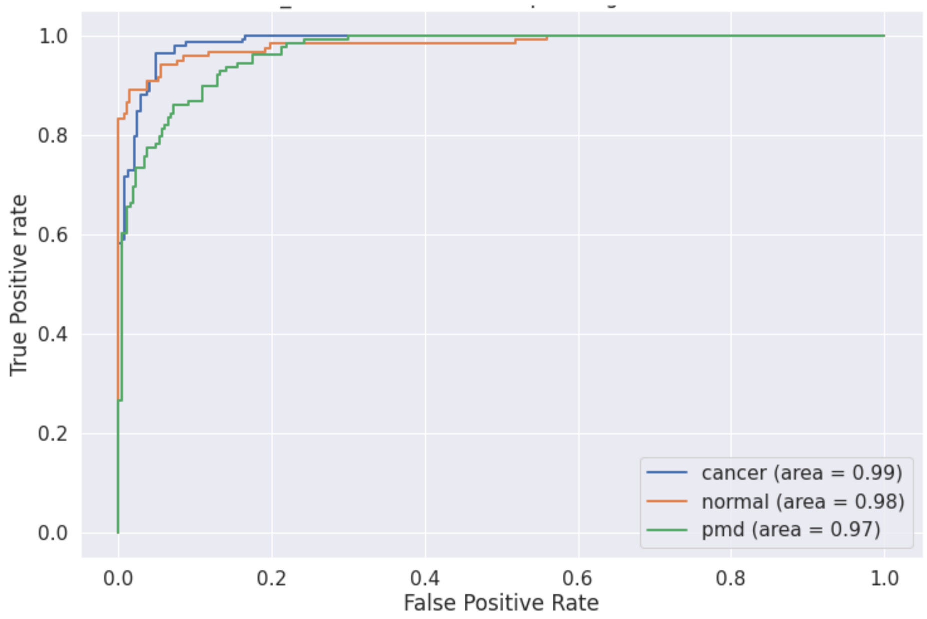

(b) ROC curve of ResNet-101
