# Supplementary material for: AI-based analysis of oral lesions using novel deep convolutional neural networks for early detection of oral cancer
Source: PLoS One. 2022 Aug 24;17(8):e0273508. doi: 10.1371/journal.pone.0273508 (PMC9401150; doi:10.1371/journal.pone.0273508)
Supplement: S2 Fig — (PDF) [file pone.0273508.s002.pdf]

S2 Fig. Normalized confusion matrix of high performance CNN-based multiclass classification models; (a) DenseNet-169, (b) ResNet-101. The y axis represents the true label, and the x axis represents the model's prediction.

pmd = Oral potentially malignant disorders.

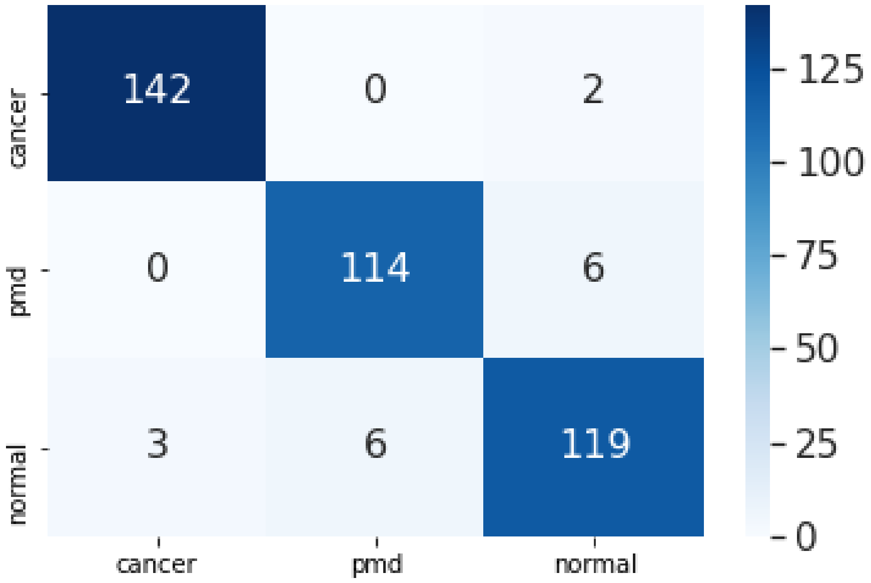

(a) Normalized confusion matrix of DenseNet-169

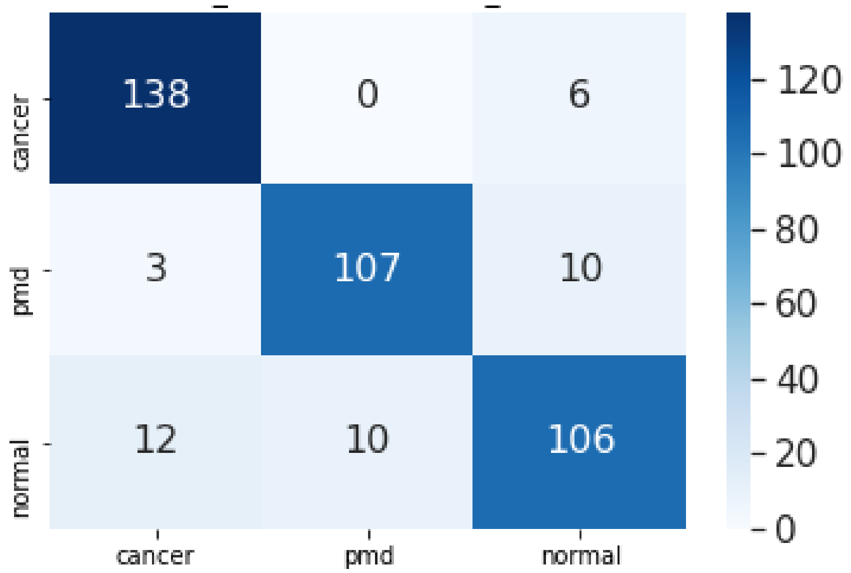

(b) Normalized confusion matrix of ResNet-101
