# Supplementary material for: AI-based analysis of oral lesions using novel deep convolutional neural networks for early detection of oral cancer
Source: PLoS One. 2022 Aug 24;17(8):e0273508. doi: 10.1371/journal.pone.0273508 (PMC9401150; doi:10.1371/journal.pone.0273508)
Supplement: S3 Fig — (PDF) [file pone.0273508.s003.pdf]

S3 Fig. **The precision-recall curve of CNN-based object detection model**; (a, b) Faster-RCNN, (c, d) YOLOv5, (e, f) RetinaNet, (g, h) CenterNet2.

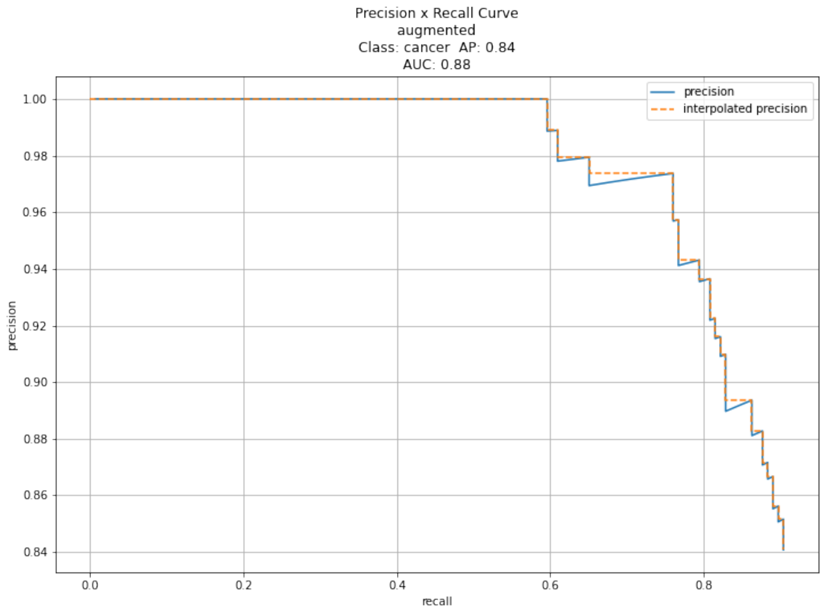

(a) Precision-Recall curve of Faster-RCNN of oral cancer

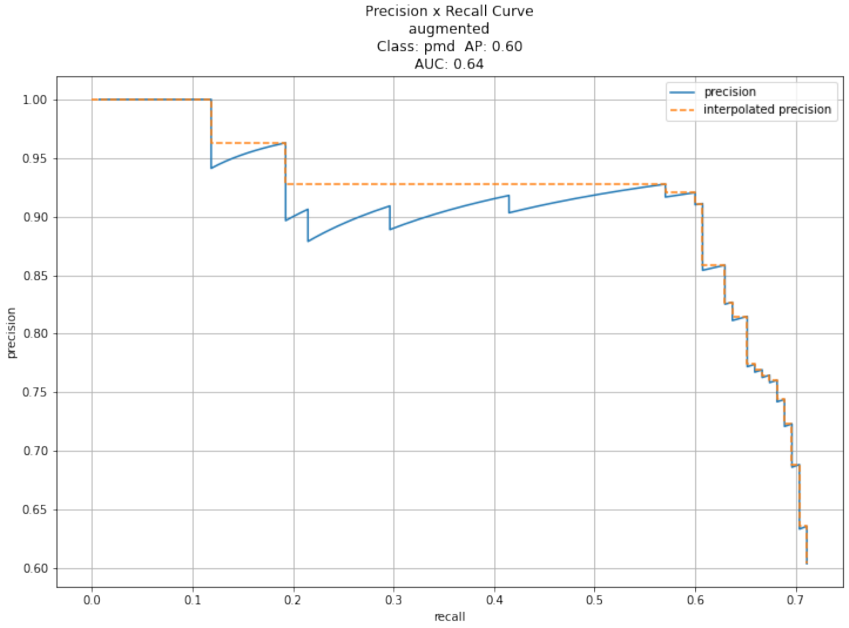

(b) Precision-Recall curve of Faster-RCNN of OPMDs

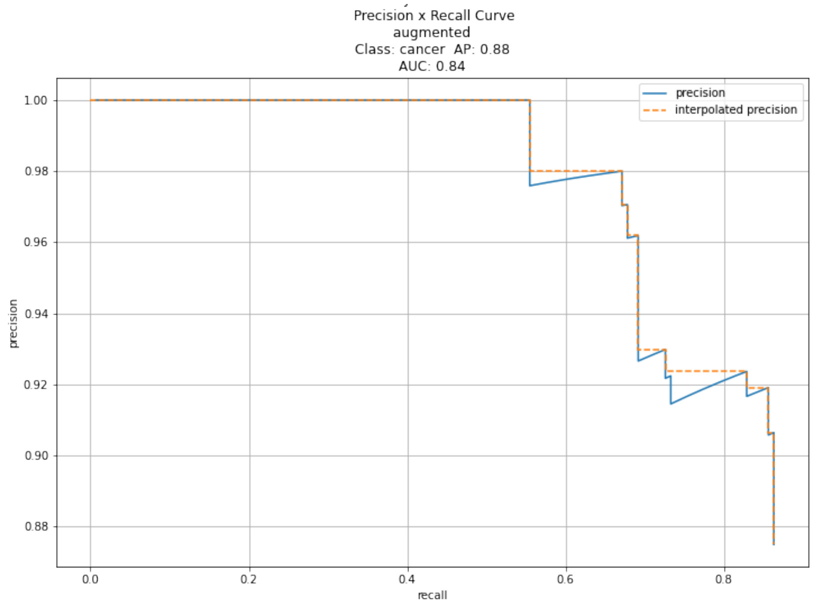

(c) Precision-Recall curve of YOLOv5 of oral cancer

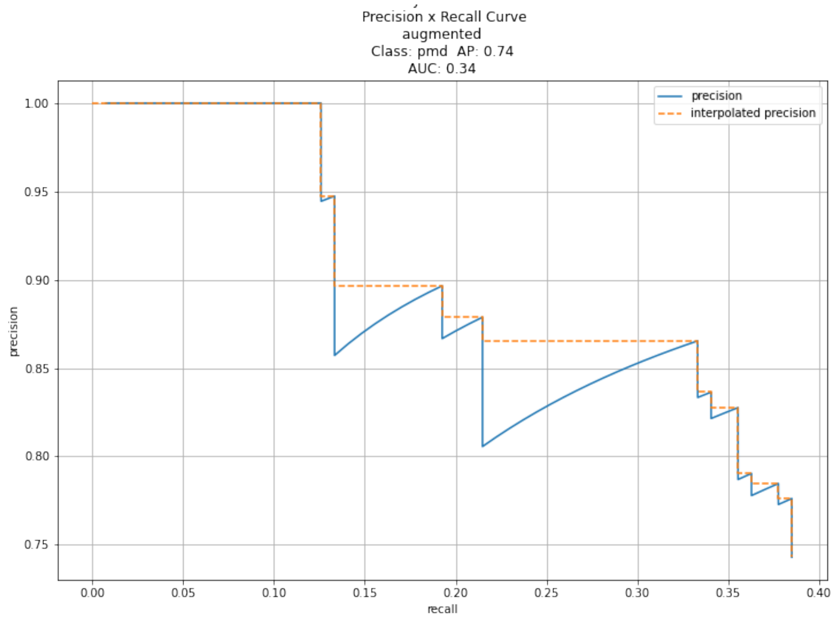

(d) Precision-Recall curve of YOLOv5 of OPMDs

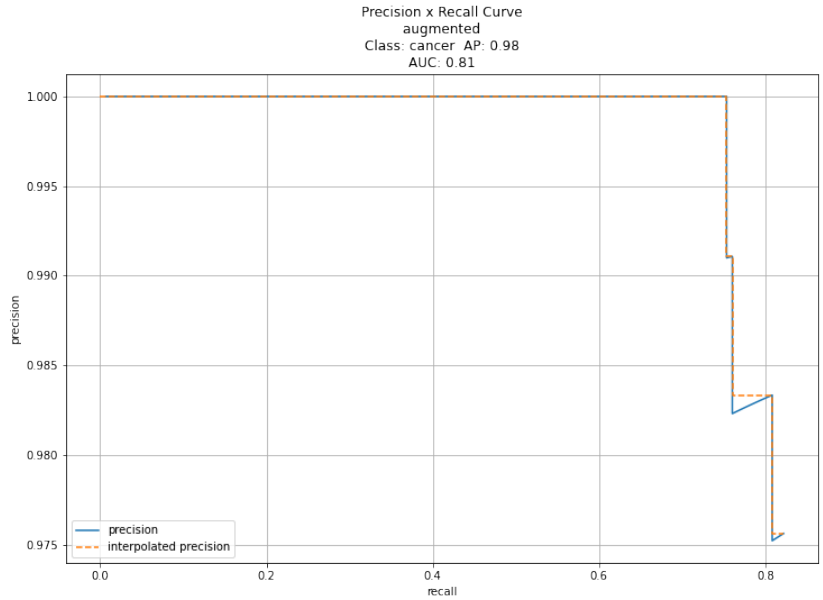

(e) Precision-Recall curve of RetinaNet of oral cancer

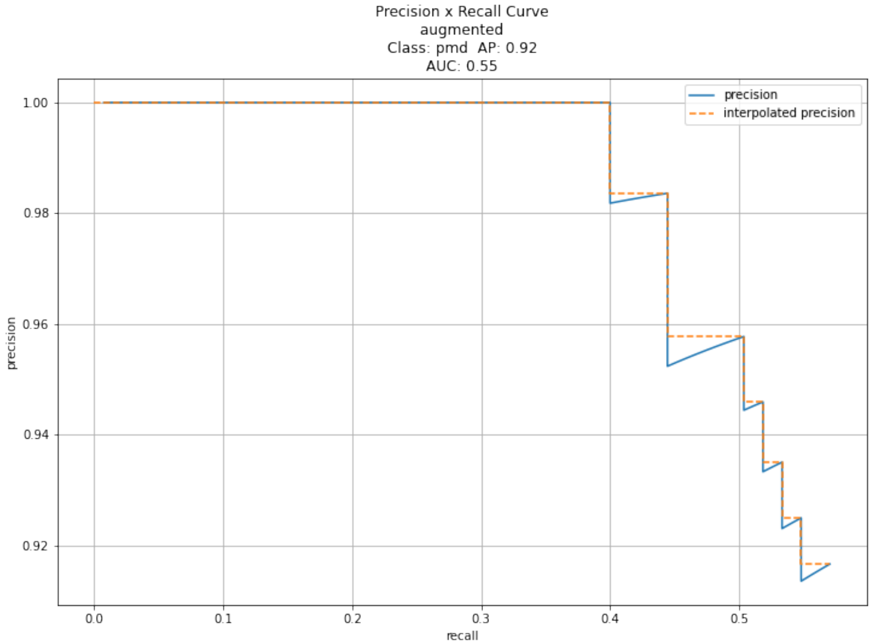

(f) Precision-Recall curve of RetinaNet of OPMDs

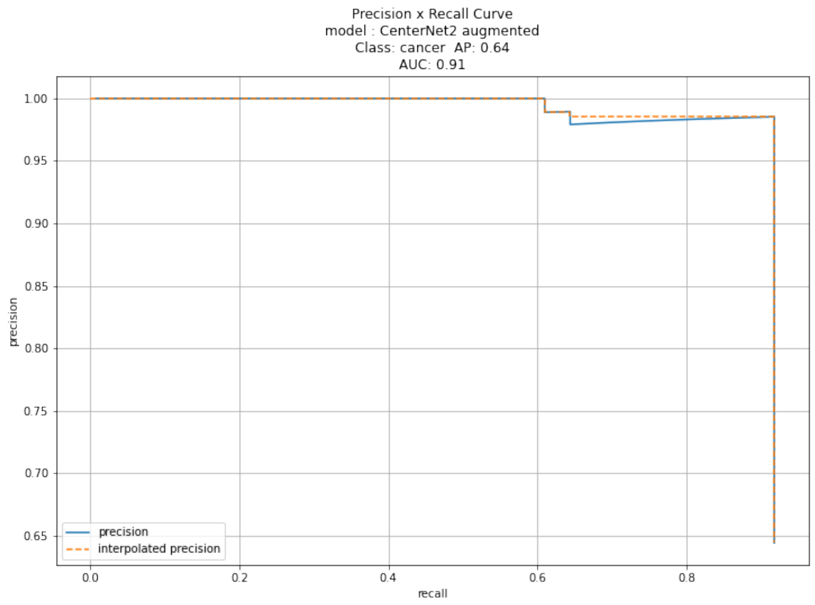

(g) Precision-Recall curve of CenterNet2 of oral cancer

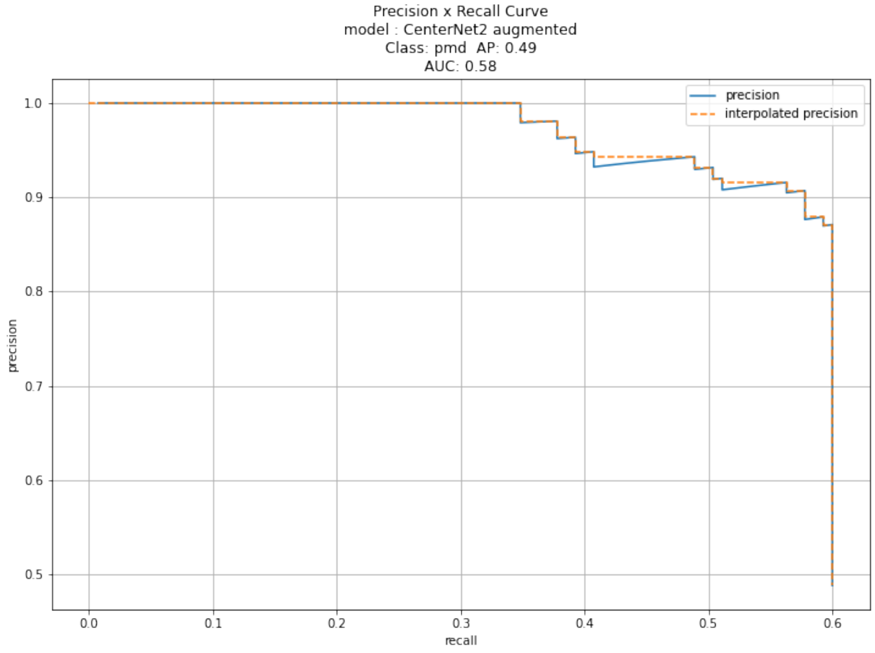

(h) Precision-Recall curve of CenterNet2 of OPMDs
