# Supplementary material for: AI-based analysis of oral lesions using novel deep convolutional neural networks for early detection of oral cancer
Source: PLoS One. 2022 Aug 24;17(8):e0273508. doi: 10.1371/journal.pone.0273508 (PMC9401150; doi:10.1371/journal.pone.0273508)
Supplement: S1 File — (PDF) [file pone.0273508.s004.pdf]

S1 File. **Object detection matrix of CNN-based object detection models.**

https://github.com/rafaelpadilla/Object-Detection-Metrics

1. **Faster R-CNN**

Class: cancer

Precision: ['1.00', '0.99', '0.99', '0.99', '0.98', '0.98', '0.98', '0.98', '0.98', '0.98', '0.98', '0.97', '0.97', '0.97', '0.97', '0.97', '0.97', '0.97', '0.97', '0.97', '0.97', '0.97', '0.97', '0.97', '0.97', '0.97', '0.97', '0.97', '0.97', '0.96', '0.96', '0.95', '0.94', '0.94', '0.94', '0.94', '0.94', '0.94', '0.94', '0.94', '0.93', '0.92', '0.92', '0.92', '0.92', '0.91', '0.91', '0.90', '0.90', '0.89', '0.89', '0.89', '0.89', '0.89', '0.89', '0.89', '0.88', '0.88', '0.88', '0.88', '0.87', '0.87', '0.87', '0.87', '0.86', '0.86', '0.86', '0.85', '0.85', '0.85', '0.84']

Recall: ['0.01', '0.01', '0.02', '0.03', '0.03', '0.04', '0.05', '0.05', '0.06', '0.07', '0.08', '0.08', '0.09', '0.10', '0.10', '0.11', '0.12', '0.12', '0.13', '0.14', '0.14', '0.15', '0.16', '0.16', '0.17', '0.18', '0.18', '0.19', '0.20', '0.21', '0.21', '0.22', '0.23', '0.23', '0.24', '0.25', '0.25', '0.26', '0.27', '0.27', '0.28', '0.29', '0.29', '0.30', '0.31', '0.32', '0.32', '0.33', '0.34', '0.34', '0.35', '0.36', '0.36', '0.37', '0.38', '0.38', '0.39', '0.40', '0.40', '0.41', '0.42', '0.42', '0.43', '0.44', '0.45', '0.45', '0.46', '0.47', '0.47', '0.48', '0.49', '0.49', '0.50', '0.51', '0.51', '0.52', '0.53', '0.53', '0.54', '0.55', '0.55', '0.56', '0.57', '0.58', '0.58', '0.59', '0.60', '0.60', '0.60', '0.61', '0.61', '0.62', '0.62', '0.63', '0.64', '0.64', '0.65', '0.65', '0.66', '0.66', '0.67', '0.68', '0.68', '0.69', '0.70', '0.71', '0.71', '0.72', '0.73', '0.73', '0.74', '0.75', '0.75', '0.76', '0.76', '0.76', '0.77', '0.77', '0.77', '0.77', '0.78', '0.79', '0.79', '0.79', '0.80', '0.81', '0.81', '0.81', '0.82', '0.82', '0.82', '0.82', '0.83', '0.83', '0.83', '0.83', '0.84', '0.84', '0.85', '0.86', '0.86', '0.86', '0.86', '0.87', '0.88', '0.88', '0.88', '0.88', '0.88', '0.89', '0.89', '0.89', '0.90', '0.90', '0.90', '0.90', '0.90']

Class: pmd

Precision: ['1.00', '1.00', '1.00', '1.00', '1.00', '1.00', '1.00', '1.00', '1.00', '1.00', '1.00', '1.00', '1.00', '1.00', '1.00', '1.00', '0.94', '0.94', '0.95', '0.95', '0.95', '0.95', '0.96', '0.96', '0.96', '0.96', '0.96', '0.93', '0.90', '0.90', '0.90', '0.91', '0.88', '0.88', '0.89', '0.89', '0.89', '0.89', '0.90', '0.90', '0.90', '0.90', '0.91', '0.91', '0.89', '0.89', '0.89', '0.90', '0.90', '0.90', '0.90', '0.90', '0.91', '0.91', '0.91', '0.91', '0.91', '0.91', '0.92', '0.92', '0.92', '0.90', '0.90', '0.91', '0.91', '0.91', '0.91', '0.91', '0.91', '0.91', '0.92', '0.92', '0.92', '0.92', '0.92', '0.92', '0.92', '0.92', '0.92', '0.93', '0.93', '0.93', '0.93', '0.92', '0.92', '0.92', '0.92', '0.92', '0.91', '0.91', '0.90', '0.89', '0.88', '0.87', '0.86', '0.85', '0.86', '0.86', '0.86', '0.85', '0.84', '0.83', '0.83', '0.83', '0.82', '0.81', '0.81', '0.81', '0.81', '0.80', '0.79', '0.79', '0.78', '0.77', '0.77', '0.77', '0.77', '0.76',

'0.76', '0.76', '0.76', '0.75', '0.75', '0.74', '0.74', '0.74', '0.73', '0.73', '0.72', '0.72', '0.72', '0.71', '0.71', '0.70', '0.70', '0.69', '0.69', '0.69', '0.68', '0.68', '0.67', '0.67', '0.66', '0.66', '0.66', '0.65', '0.65', '0.64', '0.64', '0.63', '0.64', '0.63', '0.63', '0.62', '0.62', '0.62', '0.61', '0.61', '0.60']

Recall: ['0.01', '0.01', '0.02', '0.03', '0.04', '0.04', '0.05', '0.06', '0.07', '0.07', '0.08', '0.09', '0.10', '0.10', '0.11', '0.12', '0.12', '0.13', '0.13', '0.14', '0.15', '0.16', '0.16', '0.17', '0.18', '0.19', '0.19', '0.19', '0.19', '0.20', '0.21', '0.21', '0.21', '0.22', '0.23', '0.24', '0.24', '0.25', '0.26', '0.27', '0.27', '0.28', '0.29', '0.30', '0.30', '0.30', '0.31', '0.32', '0.33', '0.33', '0.34', '0.35', '0.36', '0.36', '0.37', '0.38', '0.39', '0.39', '0.40', '0.41', '0.41', '0.41', '0.42', '0.43', '0.44', '0.44', '0.45', '0.46', '0.47', '0.47', '0.48', '0.49', '0.50', '0.50', '0.51', '0.52', '0.53', '0.53', '0.54', '0.55', '0.56', '0.56', '0.57', '0.57', '0.58', '0.59', '0.59', '0.60', '0.60', '0.61', '0.61', '0.61', '0.61', '0.61', '0.61', '0.61', '0.61', '0.62', '0.63', '0.63', '0.63', '0.63', '0.63', '0.64', '0.64', '0.64', '0.64', '0.65', '0.65', '0.65', '0.65', '0.65', '0.65', '0.65', '0.66', '0.66', '0.67', '0.67', '0.67', '0.67', '0.68', '0.68', '0.68', '0.68', '0.69', '0.69', '0.69', '0.69', '0.69', '0.70', '0.71', '0.71', '0.71', '0.71', '0.71', '0.71', '0.71', '0.71', '0.71']

2. **YOLOv5**

Class: cancer
Precision: ['1.00', '0.99', '0.98', '0.98', '0.98', '0.98', '0.98', '0.98', '0.98', '0.98', '0.98', '0.98', '0.98', '0.98', '0.98', '0.98', '0.98', '0.98', '0.98', '0.98', '0.97', '0.97', '0.96', '0.96', '0.96', '0.95', '0.94', '0.94', '0.93', '0.93', '0.93', '0.93', '0.93', '0.93', '0.92', '0.92', '0.91', '0.92', '0.92', '0.92', '0.92', '0.92', '0.92', '0.92', '0.92', '0.92', '0.92', '0.92', '0.92', '0.92', '0.92', '0.92', '0.92', '0.92', '0.92', '0.92', '0.91', '0.91', '0.91', '0.90', '0.89', '0.89', '0.88', '0.88']
Recall: ['0.01', '0.01', '0.02', '0.03', '0.03', '0.04', '0.05', '0.05', '0.06', '0.07', '0.08', '0.08', '0.09', '0.10', '0.10', '0.11', '0.12', '0.12', '0.13', '0.14', '0.14', '0.15', '0.16', '0.16', '0.17', '0.18', '0.18', '0.19', '0.20', '0.21', '0.21', '0.22', '0.23', '0.23', '0.24', '0.25', '0.25', '0.26', '0.27', '0.27', '0.28', '0.29', '0.29', '0.30', '0.31', '0.32', '0.32', '0.33', '0.34', '0.34', '0.35', '0.36', '0.36', '0.37', '0.38', '0.38', '0.39', '0.40', '0.40', '0.41', '0.42', '0.42', '0.43', '0.44', '0.45', '0.45', '0.46', '0.47', '0.47', '0.48', '0.49', '0.49', '0.50', '0.51', '0.51', '0.52', '0.53', '0.53', '0.54', '0.55', '0.55', '0.55', '0.55', '0.56', '0.57', '0.58', '0.58', '0.59', '0.60', '0.60', '0.61', '0.62', '0.62', '0.63', '0.64', '0.64', '0.65', '0.66', '0.66', '0.67', '0.67', '0.68', '0.68', '0.68', '0.69', '0.69', '0.69', '0.69', '0.69', '0.70', '0.71', '0.71', '0.72', '0.73', '0.73', '0.73', '0.73', '0.74', '0.75', '0.75', '0.76', '0.77', '0.77', '0.78', '0.79', '0.79', '0.80', '0.81', '0.82', '0.82', '0.83', '0.83', '0.84', '0.84', '0.85', '0.86', '0.86', '0.86', '0.86', '0.86', '0.86', '0.86', '0.86', '0.86']

Class: pmd
Precision: ['1.00', '1.00', '1.00', '1.00', '1.00', '1.00', '1.00', '1.00', '1.00', '1.00', '1.00', '1.00', '1.00', '1.00', '1.00', '1.00', '1.00', '0.94', '0.95', '0.90', '0.86', '0.86', '0.87', '0.88', '0.88', '0.88', '0.89', '0.89', '0.90', '0.87', '0.87', '0.88', '0.88', '0.85', '0.83', '0.81', '0.81', '0.82', '0.82', '0.82', '0.83', '0.83', '0.84', '0.84', '0.84', '0.85', '0.85', '0.85', '0.86', '0.86', '0.86', '0.87', '0.85', '0.83', '0.84', '0.82', '0.82', '0.83', '0.81', '0.80', '0.79', '0.79', '0.78', '0.78', '0.78', '0.77', '0.78', '0.76', '0.75', '0.74']
Recall: ['0.01', '0.01', '0.02', '0.03', '0.04', '0.04', '0.05', '0.06', '0.07', '0.07', '0.08', '0.09', '0.10', '0.10', '0.11', '0.12', '0.13', '0.13', '0.13', '0.13', '0.13', '0.14', '0.15', '0.16', '0.16', '0.17', '0.18', '0.19', '0.19', '0.19', '0.20', '0.21', '0.21', '0.21', '0.21', '0.21', '0.22', '0.23', '0.24', '0.24', '0.25', '0.26', '0.27', '0.27', '0.28', '0.29', '0.30', '0.30', '0.31', '0.32', '0.33', '0.33', '0.33', '0.33', '0.34', '0.34', '0.35', '0.36', '0.36', '0.36', '0.36', '0.36', '0.36', '0.37', '0.38', '0.38', '0.39', '0.39', '0.39', '0.39']

3. **RetinaNet**

Class: cancer
Precision: ['1.00', '0.99', '0.99', '0.98', '0.98', '0.98', '0.98', '0.98', '0.98', '0.98', '0.98', '0.98', '0.98', '0.98']
Recall: ['0.01', '0.01', '0.02', '0.03', '0.03', '0.04', '0.05', '0.05', '0.06', '0.07', '0.08', '0.08', '0.09', '0.10', '0.10', '0.11', '0.12', '0.12', '0.13', '0.14', '0.14', '0.15', '0.16', '0.16', '0.17', '0.18', '0.18', '0.19', '0.20', '0.21', '0.21', '0.22', '0.23', '0.23', '0.24', '0.25', '0.25', '0.26', '0.27', '0.27', '0.28', '0.29', '0.29', '0.30', '0.31', '0.32', '0.32', '0.33', '0.34', '0.34', '0.35', '0.36', '0.36', '0.37', '0.38', '0.38', '0.39', '0.40', '0.40', '0.41', '0.42', '0.42', '0.43', '0.44', '0.45', '0.45', '0.46', '0.47', '0.47', '0.48', '0.49', '0.49', '0.50', '0.51', '0.51', '0.52', '0.53', '0.53', '0.54', '0.55', '0.55', '0.56', '0.57', '0.58', '0.58', '0.59', '0.60', '0.60', '0.61', '0.62', '0.62', '0.63', '0.64', '0.64', '0.65', '0.66', '0.66', '0.67', '0.68', '0.68', '0.69', '0.70', '0.71', '0.71', '0.72', '0.73', '0.73', '0.74', '0.75', '0.75', '0.75', '0.76', '0.76', '0.77', '0.77', '0.78', '0.79', '0.79', '0.80', '0.81', '0.81', '0.82', '0.82']

Class: pmd
Precision: ['1.00', '0.98', '0.98', '0.98', '0.98', '0.98', '0.98', '0.98', '0.97', '0.95', '0.95', '0.95', '0.95', '0.96', '0.96', '0.96', '0.96', '0.96', '0.94', '0.95', '0.95', '0.93', '0.93', '0.94', '0.92', '0.92', '0.93', '0.91', '0.91', '0.92', '0.92']
Recall: ['0.01', '0.01', '0.02', '0.03', '0.04', '0.04', '0.05', '0.06', '0.07', '0.07', '0.08', '0.09', '0.10', '0.10', '0.11', '0.12', '0.13', '0.13', '0.14', '0.15', '0.16', '0.16', '0.17', '0.18', '0.19', '0.19', '0.20', '0.21', '0.21', '0.22', '0.23', '0.24', '0.24', '0.25', '0.26', '0.27', '0.27', '0.28', '0.29', '0.30', '0.30', '0.31', '0.32', '0.33', '0.33', '0.34', '0.35', '0.36', '0.36', '0.37', '0.38', '0.39', '0.39', '0.40', '0.40', '0.41', '0.41', '0.42', '0.43', '0.44', '0.44', '0.44', '0.44', '0.45', '0.46', '0.47', '0.47', '0.48', '0.49', '0.50', '0.50', '0.50', '0.51', '0.52', '0.52', '0.53', '0.53', '0.53', '0.54', '0.55', '0.55', '0.56', '0.56', '0.57']

4. CenterNet2

Class: cancer
Precision: ['1.00', '0.99', '0.99', '0.99', '0.99', '0.99', '0.99', '0.98', '0.99', '0.99', '0.99', '0.98', '0.97', '0.96', '0.96', '0.95', '0.94', '0.94', '0.93', '0.92', '0.92', '0.91', '0.91', '0.90', '0.89', '0.89', '0.88', '0.88', '0.87', '0.86', '0.86', '0.85', '0.85', '0.84', '0.84', '0.83', '0.83', '0.82', '0.82', '0.81', '0.81', '0.80', '0.80', '0.79', '0.79', '0.78', '0.78', '0.77', '0.77', '0.77', '0.76', '0.76', '0.75', '0.75', '0.74', '0.74', '0.74', '0.73', '0.73', '0.72', '0.72', '0.72', '0.71', '0.71', '0.71', '0.70', '0.70', '0.69', '0.69', '0.69', '0.68', '0.68', '0.68', '0.67', '0.67', '0.67', '0.66', '0.66', '0.66', '0.65', '0.65', '0.65', '0.64']
Recall: ['0.01', '0.01', '0.02', '0.03', '0.03', '0.04', '0.05', '0.05', '0.06', '0.07', '0.08', '0.08', '0.09', '0.10', '0.10', '0.11', '0.12', '0.12', '0.13', '0.14', '0.14', '0.15', '0.16', '0.16', '0.17', '0.18', '0.18', '0.19', '0.20', '0.21', '0.21', '0.22', '0.23', '0.23', '0.24', '0.25', '0.25', '0.26', '0.27', '0.27', '0.28', '0.29', '0.29', '0.30', '0.31', '0.32', '0.32', '0.33', '0.34', '0.34', '0.35', '0.36', '0.36', '0.37', '0.38', '0.38', '0.39', '0.40', '0.40', '0.41', '0.42', '0.42', '0.43', '0.44', '0.45', '0.45', '0.46', '0.47', '0.47', '0.48', '0.49', '0.49', '0.50', '0.51', '0.51', '0.52', '0.53', '0.53', '0.54', '0.55', '0.55', '0.56', '0.57', '0.58', '0.58', '0.59', '0.60', '0.60', '0.61', '0.61', '0.62', '0.62', '0.63', '0.64', '0.64', '0.64', '0.65', '0.66', '0.66', '0.67', '0.68', '0.68', '0.69', '0.70', '0.71', '0.71', '0.72', '0.73', '0.73', '0.74', '0.75', '0.75', '0.76', '0.77', '0.77', '0.78', '0.79', '0.79', '0.80', '0.81', '0.82', '0.82', '0.83', '0.84', '0.84', '0.85', '0.86', '0.86', '0.87', '0.88', '0.88', '0.89', '0.90', '0.90', '0.91', '0.92']

Class: pmd
Precision: ['1.00', '0.98', '0.98', '0.98', '0.98', '0.98', '0.96', '0.96', '0.96', '0.95', '0.95', '0.95', '0.93', '0.93', '0.93', '0.94', '0.94', '0.94', '0.94', '0.94', '0.94', '0.94', '0.94', '0.94', '0.93', '0.93', '0.93', '0.92', '0.92', '0.91', '0.91', '0.91', '0.91', '0.91', '0.91', '0.91', '0.92', '0.90', '0.91', '0.91', '0.90', '0.89', '0.88', '0.88', '0.88', '0.87', '0.87', '0.86', '0.85', '0.84', '0.84', '0.83', '0.82', '0.81', '0.80', '0.79', '0.79', '0.78', '0.77', '0.76', '0.76', '0.75', '0.74', '0.74', '0.73', '0.72', '0.72', '0.71', '0.70', '0.70', '0.69', '0.69', '0.68', '0.68', '0.67', '0.66', '0.66', '0.65', '0.65', '0.64', '0.64', '0.63', '0.63', '0.62', '0.62', '0.61', '0.61', '0.60', '0.60', '0.60', '0.59', '0.59', '0.58', '0.58', '0.57', '0.57', '0.57', '0.56', '0.56', '0.55', '0.55', '0.55', '0.54', '0.54', '0.54', '0.53', '0.53', '0.53', '0.52', '0.52', '0.52', '0.51', '0.51', '0.51', '0.50', '0.50', '0.50', '0.49', '0.49', '0.49']
Recall: ['0.01', '0.01', '0.02', '0.03', '0.04', '0.04', '0.05', '0.06', '0.07', '0.07', '0.08', '0.09', '0.10', '0.10', '0.11', '0.12', '0.13', '0.13', '0.14', '0.15', '0.16', '0.16', '0.17', '0.18', '0.19', '0.19', '0.20', '0.21',

'0.21', '0.22', '0.23', '0.24', '0.24', '0.25', '0.26', '0.27', '0.27', '0.28', '0.29', '0.30', '0.30', '0.31', '0.32', '0.33', '0.33', '0.34', '0.35', '0.35', '0.36', '0.36', '0.37', '0.38', '0.38', '0.39', '0.39', '0.39', '0.40', '0.41', '0.41', '0.41', '0.42', '0.43', '0.44', '0.44', '0.45', '0.46', '0.47', '0.47', '0.48', '0.49', '0.49', '0.50', '0.50', '0.50', '0.51', '0.51', '0.52', '0.53', '0.53', '0.54', '0.55', '0.56', '0.56', '0.56', '0.57', '0.58', '0.58', '0.58', '0.58', '0.59', '0.59', '0.59', '0.60']
