# Supplementary material for: AI-based analysis of oral lesions using novel deep convolutional neural networks for early detection of oral cancer
Source: PLoS One. 2022 Aug 24;17(8):e0273508. doi: 10.1371/journal.pone.0273508 (PMC9401150; doi:10.1371/journal.pone.0273508)
Supplement: Attachment — Submitted filename: Response to Reviewers.docx [file pone.0273508.s005.docx]

**Journal Requirements:**

Please review your reference list to ensure that it is complete and correct. If you have cited papers that have been retracted, please include the rationale for doing so in the manuscript text, or remove these references and replace them with relevant current references. Any changes to the reference list should be mentioned in the rebuttal letter that accompanies your revised manuscript. If you need to cite a retracted article, indicate the article’s retracted status in the References list and also include a citation and full reference for the retraction notice.

**Response:** Thanks for your suggestion, we checked the references to ensure they meet the requirements of the journal.

Additional Editor Comments (if provided):

[Note: HTML markup is below. Please do not edit.]

**Reviewers' comments:**

Reviewer's Responses to Questions

**Comments to the Author**

1. Is the manuscript technically sound, and do the data support the conclusions?

The manuscript must describe a technically sound piece of scientific research with data that supports the conclusions. Experiments must have been conducted rigorously, with appropriate controls, replication, and sample sizes. The conclusions must be drawn appropriately based on the data presented.

Reviewer #1: Yes

Reviewer #2: Yes

2. Has the statistical analysis been performed appropriately and rigorously?

Reviewer #1: Yes

Reviewer #2: Yes

3. Have the authors made all data underlying the findings in their manuscript fully available?

The [PLOS Data policy](http://www.plosone.org/static/policies.action#sharing) requires authors to make all data underlying the findings described in their manuscript fully available without restriction, with rare exception (please refer to the Data Availability Statement in the manuscript PDF file). The data should be provided as part of the manuscript or its supporting information, or deposited to a public repository. For example, in addition to summary statistics, the data points behind means, medians and variance measures should be available. If there are restrictions on publicly sharing data—e.g. participant privacy or use of data from a third party—those must be specified.

Reviewer #1: Yes

Reviewer #2: Yes

4. Is the manuscript presented in an intelligible fashion and written in standard English?

PLOS ONE does not copyedit accepted manuscripts, so the language in submitted articles must be clear, correct, and unambiguous. Any typographical or grammatical errors should be corrected at revision, so please note any specific errors here.

Reviewer #1: Yes

Reviewer #2: Yes

5. Review Comments to the Author

Please use the space provided to explain your answers to the questions above. You may also include additional comments for the author, including concerns about dual publication, research ethics, or publication ethics. (Please upload your review as an attachment if it exceeds 20,000 characters)

Reviewer #1: 1.The study presents the results of primary scientific research.
2.Results reported have not been published elsewhere.
3.Experiments, statistics, and other analyses are performed to a high technical standard and are described in sufficient detail.
4.Conclusions are presented in an appropriate fashion and are supported by the data.
5.The article is presented in an intelligible fashion and is written in standard English.
6.The research meets all applicable standards for the ethics of experimentation and research integrity.
7.The article adheres to appropriate reporting guidelines and community standards for data availability.

**Response:** We appreciated the reviewer for the comments.

Reviewer #2: Briefly try to describe the models which are used in object detection by what all layers they comprise of, and in what fashion.
If possible try to highlight the importance of one or more layers and in what way they are supportive for your case.
Also try to give some mathematical expression/equations support to your research.

**Response:** We thank the reviewer for the comment, we have added more detail on the object detection algorithms layer, more reference (ref 24) and mathematical expressions/equations in the Object detection subsection of the Materials and Methods section.

Line 181: Faster R-CNN is the very early object detection proposed to tackle both the localization and classification problems in a single deep learning network so the visual kernel can be computed once for both problems in a single deep neural network forward operation, also known as end-to-end. The input image has passed to CNN network such as VGG network to get the internal latent tensor (intermediate layer) then sends the tensor to two separate subnetworks; first subnetwork performing bounding box location regression and also computing the classification in the second subnetwork. Where the loss function is defined as
$L=$ $\frac{1}{Ncls}\sum_{i} {Lcls}_{i} + \lambda\frac{1}{Nreg}\sum_{i} {Lreg}_{i}$, where L is the total loss, i is the index of an anchor in a mini-batch, Ncls is the number of possible sub-image from sliding window, Lcls is log loss of classification, $\lambda$ is a hyperparameter to balance the two loss functions, Nreg is the number of anchor locations and Lreg is a loss function for location regression computed from the robust loss function (smooth L1) [24].

- **Reference 24:** Girshick R, editor Fast R-CNN. 2015 IEEE International Conference on Computer Vision (ICCV); 2015 7-13 Dec. 2015.

Line 196: Due to early success of Faster R-CNN in terms of high accuracy baseline, YOLO tackled another aspect of object deletion problem by dramatically increasing the frame-rate at 45 frames per second on a Titan X GPU (Nvidia Corporation, CA, USA). The intersection over union metric (IoU) is emphasized in this work to make the region proposal generation bounding box location more accurate by reframing object detection as a single regression problem, straight from image pixels to bounding box coordinates and class probabilities resulting in less computation and having high frame rate performance.

Line 206: Introduced a novel loss function by adding Focal Loss function to original cross entropy to improve accuracy of dense object detectors. Furthermore the RetinaNet architecture adopts Feature Pyramid Network (FPN), which is based on top-down pathway to allow the top level feature to laterally connect to the feature extraction of each layer leading to multi scale feature extraction capability therefore the RetinaNet able to detect smallest and biggest objects effectively.

Line 215: The CentetNet revisited the two stage object detection model, where the first stage is to compute the probability of an object in the observation image also called object likelihood to get the bounding box and the second step is to classify the object. The major difference of the CenterNet2 is applying object likelihood and conditional probability to classification $P($ $C_{k})=P(C_{k}|O_{k})P(O_{k})$, where k is index of detection bounding box $P($ $O_{k})$ is first-stage object likelihood, $P($ $C_{k}|O_{k})$ is conditional probability the given object be the class $C_{k}$ and $P($ $C_{k})$ is the probability of bounding box k be the class $C_{k}$.
